# Obligation and negative consequences in primary caregivers of dependent older relatives

**DOI:** 10.1371/journal.pone.0203790

**Published:** 2018-09-07

**Authors:** Rafael del-Pino-Casado, Catalina López-Martínez, Natalia Serrano-Ortega, Maria del Mar Pastor-Bravo, Laura Parra-Anguita

**Affiliations:** 1 School of Health Sciences, Department of Nursing, University of Jaén, Jaén, Spain; 2 University Hospital “Reina Sofía”, Córdoba, Spain; 3 School of Nursing, Department of Nursing, University of Murcia, Cartagena, Spain; 4 IMIB-Arrixaca, Murcia, Spain; Nord University, NORWAY

## Abstract

The purpose of this study was to analyse the multidimensional nature of obligation and the relations between each dimension of obligation and both anxiety and depression. A secondary analysis of data from two cross-sectional studies of primary home caregivers (N = 400; probabilistic sample) of older adult relatives in Spain was conducted. Data regarding obligation (four categories basing on beliefs of obligation and social pressure: low pressure and low beliefs, low pressure and high beliefs, high pressure and low beliefs and high pressure and high beliefs), stressors, anxiety and depression were collected by interview in 2013. The combination of high pressure and low beliefs had the highest levels of anxiety and depression, and the combination of low pressure and high beliefs had the lowest levels of anxiety and depression. When the relation of behavioural problems with anxiety and depression stratified by the previous four categories of obligation was analysed, behavioural problems were associated with anxiety and depression in the subgroups with low beliefs of obligation, whereas this association disappeared in the subgroups with high beliefs of obligation.

## Introduction

In developed countries, the increase in ageing and dependence [1] has increased the need for long-term care. In these countries, most long-term care is provided by the family [2]. Caring for an older adult dependent may have negative consequences for the caregiver’s health [3]. Such negative consequences include those related to emotional health, especially depression and anxiety [3,4].

### Factors related to anxiety and depression

Anxiety and depression are a result of the stress engendered by the caregiving activities [5]. Therefore, the theoretical models used to explain the appearance of these negative consequences of caregiving are based on the Transactional Stress Theory by Lazarus and Folkman [6]. Applying this theory to caregiving, the effects of stressors (care recipient needs) on caregivers’ health (depression, anxiety, physical health, etc.) are regulated by the caregiver’s appraisal of the caregiving situation [7].

Several authors have examined factors related with anxiety and depression in caregivers of dependent relatives. In this sense, there is evidence that the caregiver’s anxiety [8,9] and depression [4,10] are inversely related to the care recipient’s functional capacity and directly related with the care recipient’s cognitive impairment and behavioural problems.

Moreover, a few studies have analysed the factors moderating the relationships between stressors and anxiety and depression, in order to explain in more detail these relationships and enhance the prevention of these problems. The analysis of moderator factors between stressors and negative emotional consequences is supported by the above-mentioned theoretical models, and this analysis explores the existence of factors affecting the caregiver’s appraisal of the caregiving situation.

However, there has been little study on some possible factors related to anxiety and depression, as is the case of the obligation to caregiving [11]. There are few studies analysing the relation of obligation with anxiety (e.g., Losada et al. [12]) and depression (e.g., Feeney and Collins [13]), and there are no studies that analyse obligation as moderator of the relationship between stressors and anxiety and depression.

### Obligation to caregiving

The obligation to provide care can be defined as a culturally based attitude towards duty and responsibility in caring for a relative [14] and has been measured as a general obligation [15] or a filial obligation [16]. Among the several motives that lead to care for an older adult relative, obligation has attracted the interest of researchers. The reason for this attraction lies in the possible influence of obligation in the caregiving process [17].

In Lazarus and Folkman’s [6] framework, the role of culture, which is understood as the values, beliefs and rules of a particular social group, has been underrepresented [18]. Theoretical models [19,20] have been developed to integrate the cultural perspective into Lazarus and Folkman’s [6] framework. Previous models [19,20] argue that cultural factors such as motives for caregiving (obligation, reciprocity, familism) influence the caregiver’s appraisal of the caregiving situation.

Authors analysing the relation between obligation and negative caregiving consequences (anxiety, depression and burden) have found inconsistent results, finding positive associations [12,21], negative associations [11,22] or a lack of association [23,24]. This heterogeneity could be attributed to the multidimensional nature of the concept of obligation so that each dimension of obligation could be related to the negative caregiver consequences in a different manner. This hypothesis has been proposed in a previous study [17], which argues for the existence of an internal obligation and an external obligation; the internal obligation (beliefs of obligation) is related to personal beliefs about the duty of care and would protect against the negative consequences of caregiving, and the external obligation (social pressure) is related to the pressure of the social environment to provide care and would be a risk factor for these consequences. The findings of Romero-Moreno et al. [11] support the previous hypothesis. These authors found that caregivers simultaneously scoring low on internal motives for caregiving and high on external motives for caregiving may be at particular risk for negative caregiving outcomes.

The classification of the obligation for caregiving into an internal obligation (beliefs of obligation) and an external obligation (social pressure) is consistent with Ryan and Deci’s Self-determination Theory [25]. In this theory, motivation is classified into two basic kinds: intrinsic and extrinsic motivation. Intrinsic motivation refers to doing something because it is inherently valuable, interesting or enjoyable. Extrinsic motivation refers to doing something because it leads to a separable outcome, such as to satisfy an external demand or to avoid a punishment [25]. The analysis of obligation as moderator in the relationship between stressors and anxiety and depression is theoretically based on two assumptions. First, the stressors on the caregiver’s health are regulated by the caregiver’s appraisal of the caregiving situation [7]. Second, cultural factors, such as obligation to caregiving, influence this appraisal [19]. Thus, differences in obligation could lead to different effects of the stressors on the caregivers’ health in general and particularly on anxiety and depression.

Understanding the relations of the dimensions of obligation with anxiety and depression and the moderating effects of these dimensions on the relation between stressors and anxiety and depression could improve the development of a well-tailored risk profile for the prevention and early detection of anxiety and depression.

With this paper, we analyse the multidimensional nature of the concept of obligation and the relations of each dimension with anxiety and depression. Specifically, we intend to evaluate the following hypotheses:

H1: The obligation to care, measured as beliefs regarding obligation and social pressure, is related to anxiety and depression in opposite ways, so that beliefs are negatively related and social pressure is positively related.
H2: Beliefs regarding obligation and social pressure moderate the effects of the care recipient’s needs on anxiety and depression.

## Material and methods

### Design, setting and sample

Our study was a secondary analysis of data from two cross-sectional studies with samples recruited in 2013 in two Andalusian Health Districts from Jaén [26] and Córdoba [27] (Spain). The study population consisted of the primary caregivers of dependent older relatives in these Districts. This districts serves a region of 308,594 inhabitants and includes both rural and urban locations. In the two studies, frame sampling was formed by clinical records of older adult dependents that were cared for by a relative in the healthcare centres of the area (5,405 individuals). “Dependent older relatives” were considered as older relatives who are dependent in at least one activity of daily living or instrumental activity of daily living, and “primary caregiver” as the caregiver who takes responsibility for the care and gives the most amount of care. In both studies, a systematic random sampling with a sample size of 200 units was used [26,27]. So, the sample size of the secondary analysis was 400 units. Among the caregivers selected, one refused to participate in the study and was replaced by the next in the census.

A sample size of 400 units allows detection of differences in anxiety and depression among the categories of obligation with the following conditions, with a statistical significance level of 5% (calculations with PASS v. 11): 1) a minimal effect size of 0.17 for anxiety and 0.19 for depression, 2) a statistical power of 83% for anxiety and 91% for depression, for analysing differences in more than two means with the size of each category and the joint standard deviations regarding obligation shown below (in Descriptive data).

### Data collection

Data collection in the two original studies were similar [26,27]. Data were collected by interviews using questionnaires. The interviews were conducted at the care-recipient’s home in 2013 by qualified nurses (case management nurses and family nurses with at least 5–10 years of experience in caring for caregivers of disabled older relatives). The nurses had specific training in data collection (a 10-hour training session) to ensure the quality and consistency of data collection. These nurses had no previous relation with the caregivers or the care recipients. Before the interviews, caregivers were contacted by their family nurse, who informed them, during a home visit, about the study and the voluntary nature of their participation, arranging the date for the interview for those expressing an interest in participating. Privacy was ensured during the home-based interviews.

### Measures

#### Obligation

The sense of obligation as a motive for caregiving was measured by two scales previously developed [26] measuring beliefs regarding obligation (an internal dimension of obligation) and social pressure (an external dimension of obligation). Both scales (Table 1) have three Likert items with four response options (from 4 –strongly agree to 1 –strongly disagree). In the present study, Cronbach’s alpha values obtained were .75 for beliefs, .68 for pressure and .71 for both dimensions.

**Table 1:** Items used to measure beliefs regarding obligation and social pressure and their results regarding content validity (English translation from the original Spanish).

Dimension	Items	% of adequacy
Beliefs in obligation	I care for him/her because I must follow the family tradition of caring for our relatives when they cannot care for themselves	93%
	I care for him/her because, in my family, the relatives have always been cared for when they could not care for themselves.	100%
	I care for him/her because I think we all have the obligation to care for our relatives when they cannot care for themselves.	100%
Social Pressure	I care for him/her because my family and people I know expect it of me.	70%
	I care for him/her because I feel strongly forced by my family, neighbours and friends to care for my relative.	80%
	I care for him/her because my family and people I know would not approve if I took my relative to a nursing home.	93%

% of adequacy: percentage of experts considering the item as adequate to the construct measured

From the two previous scales, a new variable was created taking as cut-off point the median of each scale; this new variable had four categories: low pressure and low beliefs (LPLB), low pressure and high beliefs (LPHB), high pressure and low beliefs (HPLB) and high pressure and high beliefs (HPHB).

#### Stressors

Stressors are defined as care recipient needs and months in caregiving role [10]. The care recipient’s needs were measured by the Barthel Index (BI), the Short Portable Mental Status Questionnaire (SPMSQ) of Pfeiffer and the Neuropsychiatric Inventory (NPI) of Cummings.

The BI [28] is a 10-item scale with scores ranging from 0 to 100 that measures independence regarding the basic activities of daily living (ADL). This scale has been validated in Spain by Baztán et al. [29] with adequate psychometric properties (high criterion validity, a test-retest reliability of .98 and an inter-observer reliability of .98). The Cronbach’s alpha for the current sample was .89.

The SPMSQ [30] includes 10 items that measure cognitive impairment (range: 0–10; cognitive impairment is directly proportional to the test score). This scale has been validated in Spain by Martinez de la Iglesia et al. [31] with a sensitivity of 85.7% and a specificity of 97.3%. The Cronbach’s alpha for this study was .90.

The NPI [32] measured the frequency and severity of various behavioural problems represented by psychological and psychiatric symptoms (e.g., hallucinations, agitation, irritability, etc.). Scores range from 0 to 120 (the higher the score, the greater the frequency and severity). This has been validated in Spain by Vilalta-Franch et al. [33] with adequate psychometric properties (inter-observer reliability: .93, test-retest reliability for frequency: .79, test-retest reliability for severity: .86). The Cronbach’s alpha for the current sample was .70.

#### Negative consequences of caregiving

The negative consequences of caregiving were measured by the anxiety-depression Goldberg Scale, [34] which consists of two subscales, one for anxiety and one for depression. Each of these subscales consists of 9 questions with yes or no responses, the affirmative scoring 1 point (range of 0–9 points, the higher the score, the greater the depression or anxiety). The anxiety-depression Goldberg Scale [34] has been validated in Spain by Montón et al. [35] with good psychometric results (sensitivity 83.1%, specificity 81.8% and a positive predictive value of 95.3%). The Cronbach’s alpha for the current sample was .87 for anxiety and .89 for depression. A cut-off point of 3, as Monton et al. [35] recommended, was used for depression in the construct validity analysis of obligation.

### Ethical considerations

This study was approved by the Bioethics Committee of the University of Jaén (reference number: PI0890583) and the Research Ethics Committee of the province of Cordoba (reference number: 2809201201). All study participants signed an informed consent. Confidentiality and privacy were guaranteed throughout the study, including the publication of findings.

### Statistical analysis

For the descriptive analysis, percentages, means and standard deviations have been used. Bivariate analysis was conducted by one-way analysis of variance (ANOVA). The post-hoc analyses have been performed using the Bonferroni test. Differences of means between the categories of obligation controlling for confounders (stressors) were performed by one-way analysis of covariance (ANCOVA). The moderating effects of obligation on the relation between the care recipient’s needs and the negative consequences of caregiving have been analysed by segmenting the sample into subgroups by the variable obligation and analysing the correlation between the care recipient’s needs and the negative consequences by Pearson’s r for each subgroup. For the different statistical tests, a significance level of 0.05 was used. The calculations were performed with SPSS 15.0.

## Results

### Descriptive data

In the analysed sample, 84.5% of the caregivers were women, 60.3% were offspring and 76% shared residence with the care recipient. The average age of caregivers was 59.2 years and the average duration of caregiving was 75.8 months. These features are similar to the last Spanish national sample analysed, in which there were 83.6% of women and 61.8% of offspring [36]. No statistical differences in anxiety and depression were found by gender and kinship, and there were no statistical correlations between age and anxiety and depression. Care-recipients in the analysed sample were frail elderly with physical or mental impairments and with an average age of 82 years.

Descriptive data for the measures of the study are shown in Table 2. The most frequent categories of obligation were LPLB and LPHB. The mean of anxiety and depression were 3.7 and 3.2 (out of 9), respectively. These figure could be considered as representative of mild anxiety and depression.

**Table 2:** Descriptive data for the measures used in the study.

		Theoretical and Practical range	M	SD	No.	%
Obligation	LPLB				171	42.8
	LPHB				111	27.8
	HPLB				48	12.0
	HPHB				70	17.5
Anxiety		0–9	3.7	2.9		
Depression		0–9	3.2	2.9		
ADL		0–100	20.0	25.1		
Cognitive impairment		0–10	5.4	3.6		
Behavioural problems		0–120	10.1	13.5		
Months in caregiving role		2–600	75.8	67.9		

LPLB: low pressure and low beliefs, LPHB: low pressure and high beliefs, HPLB: high pressure and low beliefs, HPHB: high pressure and high beliefs, ADL: independence for the activities of daily living.

Descriptive data for the categories of obligation regarding beliefs of obligation, social pressure, anxiety and depression are shown in Table 3.

**Table 3:** Descriptive data (means) for the groups of obligation.

	Belief of obligation	Social pressure	Anxiety	Depression
LPLB	3.3	1.6	3.8	3.4
LPHB	8.2	1.3	2.9	2.4
HPLB	4.0	3.3	4.4	4.1
HPHB	8.6	3.5	3.9	3.1

LPLB: low pressure and low beliefs, LPHB: low pressure and high beliefs, HPLB: high pressure and low beliefs, HPHB: high pressure and high beliefs.

#### Hypothesis 1 (The obligation to care, measured as beliefs regarding obligation and social pressure, is related to anxiety and depression in opposite ways)

There were statistically significant differences in depression and anxiety among the categories of obligation (Table 4). When previous analyses were performed controlling for care recipients’ needs (one-way ANCOVA), previous differences between categories remained statistically significant (anxiety: *p* = .047, depression: *p* = .021). When months in caregiving role was added to the model, p-values for anxiety and depression remained the same (.047 and .021).

**Table 4:** Relations of categories of obligation and care recipient’s needs with anxiety and depression.

	Anxiety			Depression		
	*F* (gl)	r	p value	*F*(gl)	r	p value
Categories of obligation * ^a^	3.95 (3)		.009	4.38 (3)		.005
ADL ^b^		-.03	.51		-.07	.16
Cognitive impairment ^b^		.002	.97		.11	.036
Behavioural problems ^b^		.29	.000		.22	.000
Months in caregiving role		-.03	.52		.06	.27

* Categories of obligation: low pressure and low beliefs, low pressure and high beliefs, high pressure and low beliefs and high pressure and high beliefs; ADL: independence for the activities of daily living; F: Snedecor’s F, gl: degrees of freedom, r: Pearson’s r

^a^: one-way ANOVA test

^b^: simple linear correlation.

Post-hoc analyses (Bonferroni) showed that the level of anxiety in the category LPHB was significantly lower than the level in the category HPLB and that the level of depression in the category LPHB was significantly lower than the level in the categories LPLB and HPLB.

#### Hypothesis 2 (Beliefs regarding obligation and social pressure moderate the effects of the care recipient’s needs on anxiety and depression)

When the care recipient’s needs were related to anxiety and depression, there was a significant association between anxiety and behavioural problems, between cognitive impairment and depression and between behavioural problems and depression (Table 4).

When previous relations were analysed in each subgroup of the variable obligation (Table 5), the association between behavioural problems and anxiety and depression remained in the subgroups LPLB and HPLB whereas this association disappeared in the other subgroups; in addition, the association between cognitive impairment and depression only remained in the subgroup HPLB.

**Table 5:** Relations between care recipient’s needs and anxiety and depression in each category of obligation.

		Anxiety			Depression		
		ADL	Cognitive impairment	Behavioural problems	ADL	Cognitive impairment	Behavioural problems
Subgroups	LPHB	.11	-.13	.12	-.03	-.04	.02
	HPHB	-.10	.07	.19	.06	.13	.23
	LPLB	.021	-.001	.31**	-.156*	.14	.20**
	HPLB	.02	.24	.46**	-.14	.29*	.40**

LPHB: low pressure and high beliefs, HPHB: high pressure and high beliefs, LPLB: low pressure and low beliefs, HPLB: high pressure and low beliefs, ADL: independence for the activities of daily living.

** Correlation is significant at the .01 level.

* Correlation is significant at the .05 level.

**Fig 1:**
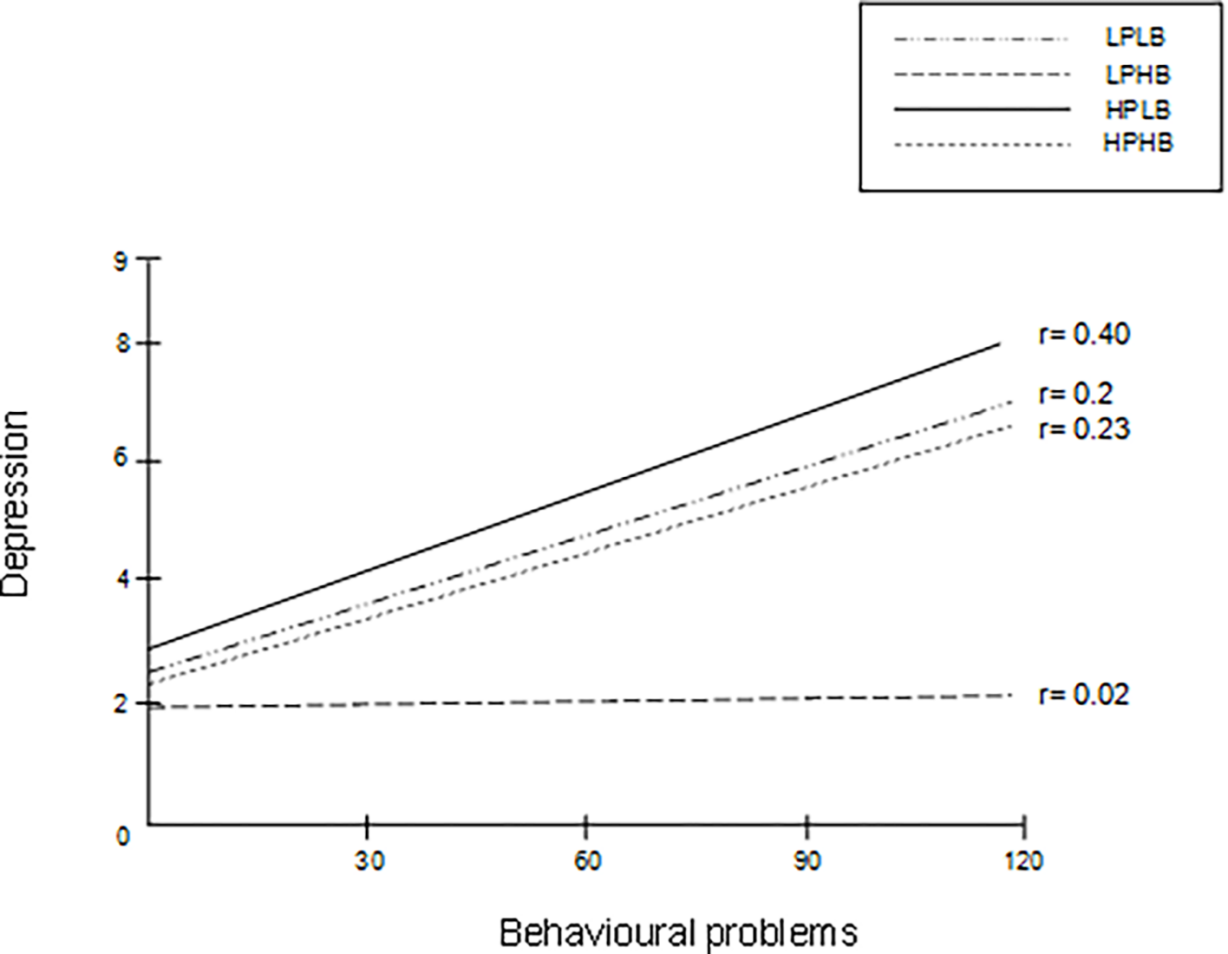
Regression lines for the relation between behavioural problems and depression in the subgroups of obligation. Notes: LPLB: low pressure and low beliefs, LPHB: low pressure and high beliefs, HPLB: high pressure and low beliefs, HPHB: high pressure and high beliefs.

**Fig 2:**
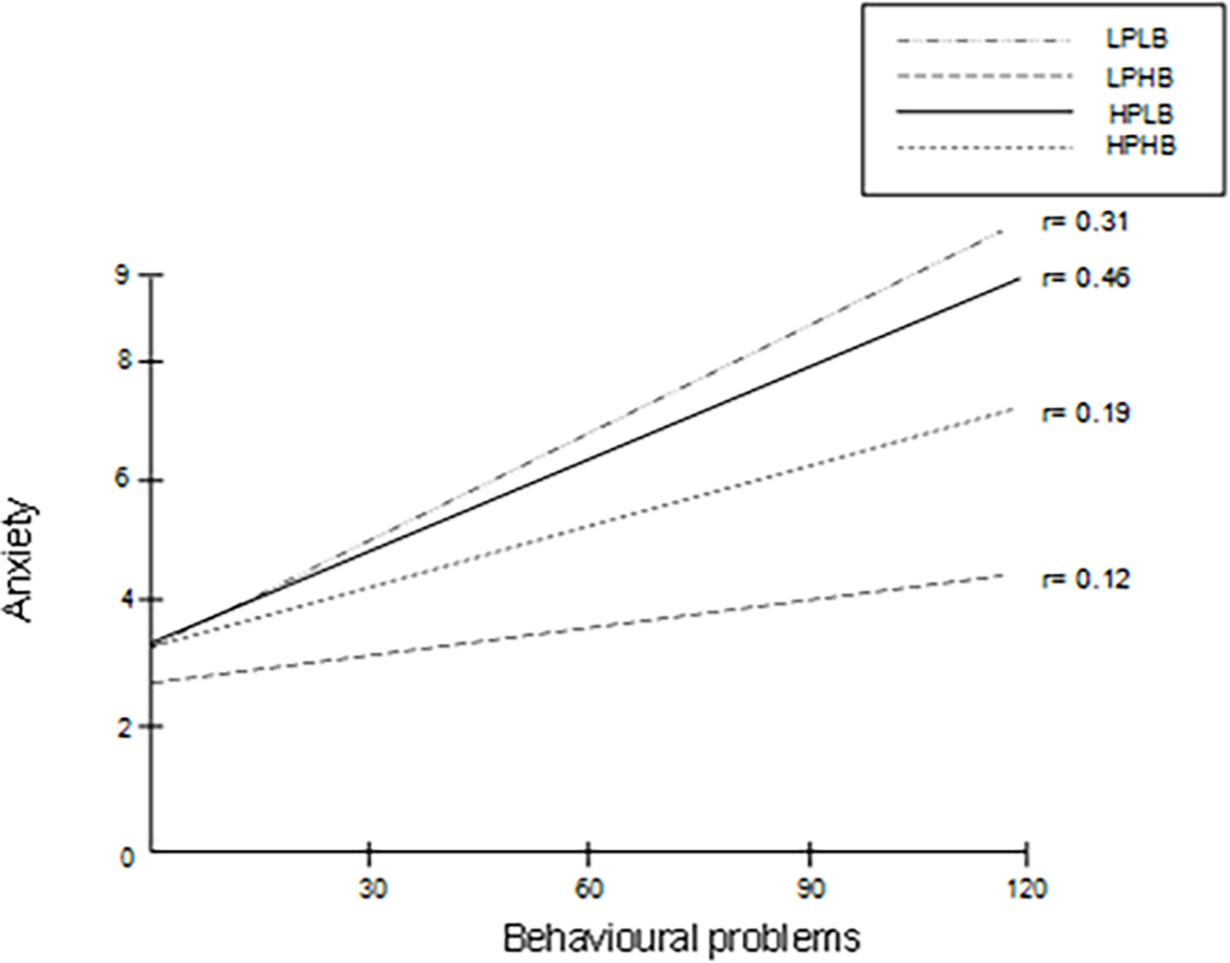
Regression lines for the relation between behavioural problems and anxiety in the subgroups of obligation. Notes: LPLB: low pressure and low beliefs, LPHB: low pressure and high beliefs, HPLB: high pressure and low beliefs, HPHB: high pressure and high beliefs.

Figs 1 and 2 show the regression line between behavioural problems and depression (Fig 1) and anxiety (Fig 2) for each subgroup. Notably, in the subgroup LPHB, behavioural problems had no effect on depression (Fig 1) and had little effect on anxiety (Fig 2).

## Discussion

In the present study we found that the obligation to caregiving, measured by the combination of belief in obligation and social pressure, was related to anxiety and depression in opposite ways. Thus, the combination HPLB had the highest levels of anxiety and depression, and the combination LPHB had the lowest levels of anxiety and depression. Furthermore, when analysing the relation between the care recipient’s needs and the negative consequences of caregiving (anxiety and depression) across the subgroups of obligation, there was no relation between behavioural problems and the negative effects in the subgroups with high beliefs (LPHB and HPHB) whereas the subgroup HPLB was the one in which cognitive impairment and depression were related.

This study has the following strengths: 1) the probabilistic nature of the sample prevents the onset of selection biases associated with convenience samples, which are quite common in the literature on informal care [37]; 2) the use of a sufficient sample size prevents the onset of type II errors.

### Hypothesis 1

Our results supported hypothesis H1 regarding the relation between obligation and anxiety and depression. Our results agreed with those of Romero-Moreno et al. [11] and, because each of the four categories of obligation is differently related to anxiety and depression, supported the multidimensionality of the concept of obligation. Furthermore, because the combination HPLB had the highest levels of anxiety and depression and the combination LPHB had the lowest levels of anxiety and depression, our findings may support that internal motives for caregiving protect one from the negative consequences of caregiving and that external motives increase the risk of such consequences. Thus, caregiving that was perceived as a personal choice rather than an externally opposed obligation could have fewer negative consequences than the opposite situation.

Our findings regarding two dimensions in obligation could be explained as following Ryan and Deci’s [25] theory of self-determination because the external motives may not be considered valuables by the individuals and may not be related to personal values and beliefs. In relation to the sense of obligation, the risk profile for anxiety and depression could be represented by caregivers with weak beliefs in obligation and a strong sense of social pressure for caregiving.

Our findings also helped explain the heterogeneity appearing when obligation and the negative consequences of caregiving are related [17]. In this sense, it is possible that the dimensions of obligation vary from one study to another and, because each dimension of obligation may be related in a different manner to a particular negative consequence of caregiving, the relation between obligation and this particular consequence vary from one study to another.

Given the above, we think that the different dimensions of obligation studied here could be detected early through targeted screening and, through the relation of obligation with anxiety and depression, this screening could help to identify individuals who need intervention to prevent anxiety and depression, in primary caregivers of older adult relatives.

Our results also supported the complexity of the concept of obligation for caregiving because, like Romero-Moreno et al. [11], we found people with both low and high scores on beliefs and pressure simultaneously. The first situation (low scores) could be explained by the fact that there are people whose motives for caregiving are unrelated to obligation but are related to other aspects such as reciprocity or affection [17]. The second situation (high scores) shows that external and internal dimensions of obligation are compatible. This situation justifies considering both dimensions in the measurement of obligation.

### Hypothesis 2

Regarding hypothesis H2 (the moderating effect of the obligation on the relation between the care recipient’s needs and the negative consequences of caregiving), our data supported this hypothesis for the relation between cognitive impairment and depression and for the relation between behavioural problems and anxiety and depression. In our findings, behavioural problems had no effect on anxiety and depression in the subgroups with strong beliefs; therefore, strong beliefs of obligation may buffer the effect of behavioural problems on anxiety and depression. These findings show that when caregiving is accepted as a personal choice based on personal values and not as an obligation imposed externally, it may generate fewer negative effects on the emotional health of the caregiver.

On the other hand, our findings also showed that the association between cognitive impairment and depression only remained in the subgroup HPLB, being the subgroup that had the most negative effects. These data are related to the data mentioned above in the discussion of hypothesis H1 regarding the relation between obligation and the negative consequences of caregiving.

Caring for a dependent older relative may be a hard and stressful task [3]. Being a caregiver involves changes and sacrifices in order to meet the needs of the dependent older adult [15]. The level of stress depends not only on the caregiving tasks but also the appraisal of the caregiving situation [7]. Our findings support the idea that motives for caregiving influence the perception of the caregiving situation and therefore may influence the caregiving consequences. Caregivers with internal motives could have less negative consequences than caregivers with external motives and this issue may be useful for the prevention of caregiving negative consequences.

### Limitations

This study has the limitation of being cross-sectional. This limitation does not allow for establishing causal relations or conclusions about changes over time in the variables studied. More research is required to properly establish the temporal sequence between beliefs regarding obligation and social pressure and the negative consequences of caregiving.

## Conclusions

Our findings supported the importance of analysing caregiver obligation in relation to care of a dependent older relative. Although obligation has usually been studied in caregiving as a unique dimension, our findings supported the existence of two dimensions: an external obligation represented by the social pressure and an internal obligation represented by beliefs of obligation. As we expected in our hypothesis, both dimensions of obligation (social pressure and beliefs of obligation) seem to relate with depression and anxiety in a different manner, so that social pressure may be a risk factor for anxiety and depression whereas beliefs regarding obligation may protect from anxiety and depression. In addition, our findings supported that strong beliefs of obligation may buffer the effect of behavioural problems on anxiety and depression. Last, our results showed the complexity of the concept of obligation because we found people with both low and high scores on beliefs regarding obligation and social pressure simultaneously.

The above findings support the following recommendations for nursing practice: 1) it may be convenient to take into account the obligation for caregiving in the nursing assessment of primary caregivers of dependent older relatives; namely, this assessment should discriminate between internal obligation (beliefs) and external obligation (social pressure); 2) to improve prevention and early detection of depression and anxiety in this population, a risk profile could be developed to identify caregivers with low beliefs in obligation and high feelings regarding social pressure. Moreover, the multidimensionality of the concept of obligation should be taken into account in the research regarding obligation for caregiving.
